# Patterns and predictors of recurrence after open radical cystectomy for bladder cancer: a comprehensive review of the literature

**DOI:** 10.1007/s00345-017-2115-4

**Published:** 2017-11-16

**Authors:** Andrea Mari, Riccardo Campi, Riccardo Tellini, Giorgio Gandaglia, Simone Albisinni, Mohammad Abufaraj, Georgios Hatzichristodoulou, Francesco Montorsi, Roland van Velthoven, Marco Carini, Andrea Minervini, Shahrokh F. Shariat

**Affiliations:** 1 Department of Urology, University of Florence, Careggi Hospital, Florence, Italy; 2 Department of Urology, Medical University of Vienna, Vienna, Austria; 3 Division of Oncology/Unit of Urology, IRCCS San Raffaele Hospital, URI, Milan, Italy; 4 Department of Urology, Institut Jules Bordet, Université Libre de Bruxelles, Bruxelles, Belgium; 5 Division of Urology, Department of Special Surgery, Jordan University Hospital, The University of Jordan, Amman, Jordan; 6 Department of Urology and Pediatric Urology, Julius-Maximilians-University of Würzburg, Würzburg, Germany; 7 Karl Landsteiner Institute of Urology and Andrology, Vienna, Austria; 8 Department of Urology, University of Texas Southwestern Medical Center, Dallas, TX USA; 9 Department of Urology, Weill Cornell Medical College, New York, NY USA; 10 Department of Urology and Comprehensive Cancer Center, Vienna General Hospital, Medical University of Vienna, Währinger Gürtel 18-20, 1090 Vienna, Austria

**Keywords:** Bladder cancer, Recurrence, Radical cystectomy, Lymph node dissection, Neoadjuvant chemotherapy, Adjuvant chemotherapy

## Abstract

**Purpose:**

To review the currently available literature reporting the patterns of recurrence and their predictive factors after open radical cystectomy (RC) for bladder cancer.

**Methods:**

A review of the literature was performed using the MEDLINE, Scopus and Web of Sciences databases from January 1997 to May 2017. The PRISMA guidelines were followed for the conduct of the study.

**Results:**

Local recurrence rate ranges between 30 and 54%. Distant recurrence is not often standardized and is reported in up to 50% of cases. The overall 5-year recurrence-free survival rates from 58 to 81%. The mean follow-up of studies included in the analysis ranged from 18 to 350 months. Details on the most important demographic and epidemiological, clinical, histologic and pathologic predictors of recurrence after radical cystectomy are provided through an evidence-based approach. The impact of the extension of lymph node dissection on recurrence after RC is investigated.

**Conclusions:**

A correct prognostic assessment is essential for patients undergoing radical cystectomy for bladder cancer, thereby potentially improving their oncologic outcomes.

**Electronic supplementary material:**

The online version of this article (10.1007/s00345-017-2115-4) contains supplementary material, which is available to authorized users.

## Introduction

Bladder cancer (BC) is the 9th most commonly diagnosed cancer and is ranked 13th for cancer deaths in the overall population in 2015 worldwide. Approximately 30% of newly diagnosed bladder cancer patients present with muscle-invasive BC (MIBC) disease with 10–15% of cases already metastatic at diagnosis. The risk of progression for high-risk non-MIBC after 5 years is 45% [1]. The standard treatment for patients with clinically localized (cT2-T4a N0M0) MIBC is radical cystectomy (RC) with bilateral pelvic lymph node dissection (PLND). The use of neoadjuvant chemotherapy (NAC) may be a valid option for MIBC but its benefit is still debated. In addition, it is reasonable to propose immediate RC to those patients with non-MIBC at highest risk of progression. Furthermore, early RC should be offered to all patients with non-MIBC failing endovesical therapy [1].

Unfortunately, a significant proportion of patients with MIBC experiences recurrence and eventually death after RC. There is no definitive evidence regarding the recurrence rate after RC. In particular, the definition of local and distant recurrence is not standardized, the recurrence-free survival (RFS) is highly variable and the specific timing of recurrence is not well defined across the published series [2–5]. Several factors have been variously associated with recurrence after RC. However, the different patient selection and the variable use of preoperative treatments among studies could affect the impact on the analysis of predictors of recurrence after RC.

Under this light, we aimed to review the currently available literature reporting the patterns of recurrence and their predictive factors after open RC. Moreover, we critically assessed the current limitations of the published series outlining potential implications for future research.

## Materials and methods

**Fig. 1:**
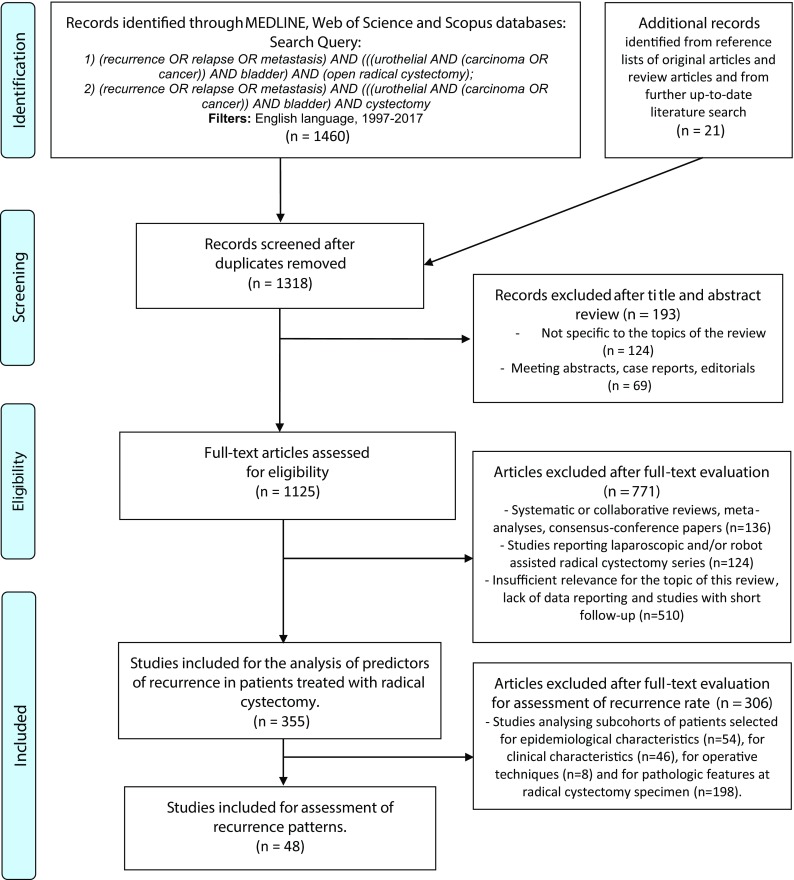
Flow chart for article selection process

A comprehensive review of the English-language literature was performed using the MEDLINE, Scopus and Web of Sciences databases from January 1st, 1997 to May 1st, 2017 following the preferred reporting items for systematic reviews and meta-analyses (PRISMA) statement using the following keywords: “recurrence” or “relapse” or “metastasis” and “bladder cancer” or “urothelial bladder cancer” and “radical cystectomy” (Fig. 1). After a first screening based on study title and abstract, all papers were assessed based on full text and excluded with reasons when appropriate. Two reviewers (A.M. and R.C.) carried out this process independently. Disagreement was solved by a third party (G.H.). The list of articles that were judged to be highly relevant by these authors was reviewed by all coauthors until a final consensus was reached on the articles included in the analysis. The “risk-of-bias” evaluation of the studies included to investigate the recurrence rate (*n* = 48) was assessed according to the Cochrane Handbook for Systematic Reviews of Interventions [6].

## Evidence synthesis

A total of 1481 articles were identified. After exclusion of duplicates, case reports, congress abstracts, editorials and papers with topics that were not specific for this review, 1125 papers were selected for full-text assessment of eligibility. After full-text evaluation, 355 studies were included for the analysis of predictors of recurrence in patients treated with radical cystectomy. Of these, 48 studies were included for the assessment of recurrence rate (Fig. 1).

### Type of studies and quality of the evidence

For the analysis of the predictors of recurrence, 313 studies were retrospective, 14 were prospective and 27 were randomized clinical trials (RCTs). Sample size ranged between 33 and 9064 patients. Most series involved multiple surgeons. For the analysis of recurrence rate, 29 studies were retrospective, 3 were prospective and 16 were RCTs. Sample size ranged between 110 and 9064 patients. Risk of bias evaluation is summarized in Supplementary Table 1.

### Definition and controversies

EAU Guidelines defined local recurrence after RC as any recurrence that takes place in soft tissues at the original surgical site or lymph nodes (LN) in the area of PLND [1]. Data in literature are very inconsistent, as a detailed discrimination between local and distant recurrence is not always present. Similarly, the percentages of RFS are highly variable even across large surgical series. Moreover, some studies include different neoadjuvant or adjuvant chemotherapy (AC) regimens, which may hinder the overall interpretation of surgical series.

### Recurrence rates in patients treated with open RC

In a retrospective study involving 4118 patients naïve of NAC treated at several institutions, the RFS was 63, 60 and 57% at 3, 5 and 10 years, respectively [2]. Xylinas et al. [3] analyzed the oncologic outcomes of 2145 patients naïve of NAC treated at European and US institutions with open RC for pT1-T3N0 urothelial BC. The RFS was 68% at a median follow-up of 48 months. Similarly, Zehnder et al. reported the oncologic outcomes of 1488 patients at a median follow-up of 162 months [4]. Overall, 6% received NAC and 3% preoperative radiotherapy (RT). The 5- and 10-year RFS were 68 and 64%, respectively, with a time to recurrence ranged from 0 to 29.2 years.

The recurrence rates in patients treated with NAC and open RC are still undetermined. In most of the retrospective studies, a low percentage of patients receive NAC due to the contraindications and the underuse reported in several centers. The best shreds of evidence come from RCTs, even if the real number of patients effectively submitted to the combined therapy is difficult to estimate due to the variable number of patients declining NAC and of those not completing the full number of cycles for adverse events.

In the experimental arm from the BA06 30894 (International Collaboration of Trialists) RCT, 491 BC patients with T2-4N0-xM0 stage underwent NAC (cisplatin, methotrexate, and vinblastine) and radical treatment (50% RC, 42% radical RT and 8% preoperative RT plus RC) [7]. The median locoregional disease-free and metastasis-free survival for the group were 23 months (47% at 3 years) and 32 months (45% at 3 years). In the Southwest Oncology Group (SWOG) RCT, 153 patients with T2-4N0M0 stage BC were randomly assigned to methotrexate, vinblastine, doxorubicin, and cisplatin (MVAC) NAC and subsequent RC [8]. At 5 years, 57% of the patients were alive. After a median follow-up of 8.7 years, 35% of patients died of disease. In a meta-analysis, the oncologic outcomes of 2491 patients treated with NAC and radical treatment (RC, radical RT, or preoperative RT and RC) from 10 clinical RCTs were reported. The locoregional disease-free survival was 166/217 (76.5%) and 1232/1963 (62.8%) in patients treated with single agent platinum and platinum-based combination, respectively. The metastasis-free survival rate was 154/217 (71%) and 1181/1963 (60.2%) in patients treated with single agent platinum and platinum-based combination, respectively.

According to the studies included in this review, the reported local recurrence rate after open RC was between 29.9 and 53.8%, and the 5-year RFS rates from 58 to 80.9%. The median follow-up of studies included in the analysis ranged from 18 to 350 months.

### Predictive factors of recurrence-free survival in patients treated with open RC

#### Demographic and epidemiologic characteristics

Although men are three to four times more likely to develop BC, several epidemiologic reports associated female gender with a more advanced disease and worse survival rates [9, 10]. Messer et al. [11] confirmed that female gender, after adjusting to standard clinical and pathologic features, was associated with an increased risk for cancer-specific mortality (CSM) and disease recurrence compared to male gender in patients undergoing open RC. In other studies, gender was not associated with outcomes in stage-adjusted analyses, whereas pathologic tumor stage remained the most powerful factor influencing the course of disease in both genders [12].

Elderly patients have more years to compound comorbidities. As such, they are associated with higher mortality after RC [13]. In a retrospective analysis of 605 patients treated with open RC, Horovitz et al. reported that octogenarians had comparable RFS to the younger counterparts [14]. Conversely, an overview of the Bladder Cancer Research Consortium reported that patients aged > 80 years had a significantly greater risk of disease recurrence than patients aged ≤ 60 years [15]. However, the retrospective nature of the studies suffered of the selection bias related to the higher use of bladder-sparing techniques in older patients and to the lower use of both NAC and AC.

In a retrospective multicentre study on more than 1500 patients, current smoking status and high cumulative smoking exposure at the time of RC was significantly associated with advanced tumor stage, nodal metastasis, disease recurrence, and CSM in patients treated with RC for high-risk non-MIBC and MIBC [16]. Smoking cessation > 10 years prior to RC mitigates the detrimental effect of smoking. Conversely, in two different studies, smoking was not an independent predictor of RFS [17, 18]. In addition, smoking seemed to affect survival only among men, but not in women, who had overall a poorer survival compared to the male gender [17]. The most important studies of this paragraph are summarized in Table 1.

**Table 1:** Selected studies analyzing the demographic and epidemiologic predictive factors of recurrence in patients treated with open radical cystectomy for bladder cancer

First author and year	Number of patients	Median age (years)	NAC (%)	Clinical tumor stage	Pathologic tumor stage	Pathologic node stage	Adjuvant therapies (%)	Median follow-up (months)	Survival	Predictive factors associated with recurrence and other findings
Messer et al. [11], 2014	4216	NR	0	NR	pT0-1 + pTis: 31.3% pT2: 24.1% pT3-4: 44.6%	pN+: 25.5%	23.5	31.5	5-year RFS (female cohort): 59.2% 5-year RFS (male cohort): 63.3%	After the adjustment for standard clinical and pathologic features, **female gender (HR 1.27;** ***p*** **=** **0.007)** was associated with an increased risk of disease **recurrence** compared with male gender
Mitra et al. [12], 2014	828	< 65 years: 45% > 65 years: 55%	5.2	NR	pT0/pTa/pTis: 20.8% pT1: 14.5% pT2: 23.7% pT3: 35% pT4: 6%	pN+: 25.6%	20	146	5-year RFS: 57%	**Increasing pathologic stage (** ***p*** **=** **0.040) and positive surgical margins (HR: 4.02;** ***p*** **=** **0.007)** were independently associated with higher risk of **recurrence**
Horovitz et al. [14], 2013	605	< 60 years: 27% 60–69 years: 32% 70–79 years: 33% ≥ 80 years: 7.8%	9.2	CIS/cTa: 14.4% cT1: 28.1% cT2: 44.8% cT3: 8% cT4: 4.7%	pT0: 21.8% pT1: 16.2% pT2: 21.8% pT3: 28.6% pT4a: 10% pT4b: 1.6%	pN0: 52.9% pN1: 12.2% pN2: 7.8% pN3: 0.5% pNx: 26.6%	12.4	26	NR	Pathological stage (*p* < 0.001) and LN positivity (HR: 2.1; *p* < 0.001) were the only variables that proved significant in multivariate analysis for RFS while **age group was not a significant factor** (*p* = 0.9)
Nielsen et a. [15], 2007	888	66	4	cTa: 2% cTis: 11% cT1: 24% cT2: 52% cT3: 6% cT4: 5%	pT0: 7% pTa: 3% pTis: 12% pT1: 13% pT2: 23% pT3: 30% pT4: 11%	pN+: 23%	24	39	3-year RFS: 62.3% 7-year RFS: 54.5% 10-year RFS: 52.3%	**Patients older than 80 year (HR: 1.74;** ***p*** **=** **0.044)** had a **significantly higher risk of disease recurrence** than patients younger than 60 year
Rink et al. [16], 2013	1506	66.4	0	NR	pT0: 5.2% pTa: 4.1% pTis: 11.2% pT1: 11.3% pT2: 26.6% pT3: 30.5% pT: 4 11.2%	pN+: 22%	21.4	34	3-year RFS: 94% 10-year RFS: 82%	**Current smoking status (HR: 1.47;** ***p*** **=** **0.020) and high cumulative smoking exposure (HR: 1.26;** ***p*** **=** **0.020) at time of RC** were independent predictors of disease **recurrence**
Bostrom et al. [17], 2012	546	Smokers: 65 years Non-smokers: 68 years	NR	≤ T2: 86% ≥ T3: 14%	≤ pT1: 39% pT2: 21% pT3: 28% pT4: 11%	pN+: 20%	NR	NR	NR	NR

Bold highlights the risk related to recurrence for each of the factors included

*CIS* carcinoma in situ, *NAC* neoadjuvant chemotherapy, *NR* not reported, *RFS* recurrence-free survival

#### Clinical characteristics

Several serological parameters have been investigated as possible predictors of recurrence in patients treated with open RC for MIBC. The pre-treatment neutrophil-to-lymphocyte ratio (NLR) is an emerging marker of host inflammation. Several studies reported the impact of NLR on oncologic outcomes in patients undergoing RC. The threshold varied among studies (from 2, 7 to 3). NLR was significantly associated with extravesical tumor extension, node involvement and was an independent predictor of relapse and CSM after RC [19, 20]. Similarly, the lymphocyte-to-monocyte ratio (LMR), an alternative marker of inflammation, was significantly associated with RFS [19, 21]. Preoperative hypoalbuminemia (< 3.5 g/dL) [22] and low albumin/globulin ratio (≥ 1.60) [23] were found to be independent predictors of RFS and OS, and RFS and cancer-specific survival (CSS), respectively. Moreover, the low albumin/globulin ratio remains an independent predictor of RFS and CSS even in a subcohort of patients with normal serum albumin [23].

In a retrospective analysis of 906 treated with RC, thrombocytosis (defined as > 400,000 platelets/mcl) was significantly associated with adverse pathologic disease stage and LN involvement [24]. Thrombocytosis was independently associated with OS, but not with RFS, after adjusting for clinicopathological factors.

A recent meta-analysis analyzed 11 and 7 studies evaluating the impact of anemia and hemoglobin level as continuous variable, respectively, in patients treated with RC. Both anemia and hemoglobin level were independently associated with increased all-cause mortality, CSM and disease recurrence [25].

The AB0 blood group antigen was found to be associated with a higher tumor stage, but not a higher risk of relapse in patients treated with RC [26]. In a subanalysis of patients treated with AC after RC, the AB blood group antigen expression was significantly associated with a worse CSM compared to the A0, B0 and 00 blood groups [26].

The current evidence supports the prognostic role of inflammatory blood-based markers and physical characteristics on oncologic outcomes after RC. Indeed, systemic inflammation seems to play a critical role in the pathogenesis of malnutrition. In a restricted cohort of 117 Japanese patients, the prognostic nutritional index (PNI) but not the controlling nutritional status (CONUT) was significantly associated with disease-specific survival after RC [27]. However, further studies are necessary to demonstrate the association between the preoperative nutritional status and oncologic outcomes in patients treated with RC for BC.

Patients with comorbidities are often less likely to have PLND and NAC [28]. Several studies reported that a higher comorbidity score was significantly associated with lower OS, but not RFS in patients undergoing open RC [29, 30]. However, after developing a recurrence, the presence of comorbidities was shown to have an independent correlation with lower survival [30]. A retrospective study including 4118 patients treated with open RC showed that obesity was significantly associated with higher tumor grade, soft-tissue surgical margins (STSMs) and worse oncologic outcomes [2]. Other authors reported that a body mass index > 30 is an independent predictor of disease recurrence and CSM [31].

In patients with diabetes mellitus undergoing RC, diabetes mellitus was significantly associated with worse CSS [32]. Moreover, metformin use was related to a decreased risk of disease recurrence and CSM [32].

Clinical staging at transurethral resection (TUR) is a fundamental diagnostic and therapeutic tool for patients with BC [1, 33]. However, the clinical stage at TUR was shown to be divergent from pathologic tumor stage in more than a half of the reported cases [34]. Patients with clinical understaging were associated with a higher risk of LN involvement at RC specimen and with worse CSM after RC [34].

In a retrospective evaluation of 297 patients treated with open RC without NAC, Culp et al. identified the presence of micropapillary or neuroendocrine features together with lymphovascular invasion (LVI) at TUR specimen, hydroureteronephrosis and cT3b-T4a disease as readily assessable clinical factors to define a high-risk disease [35]. Patients with high-risk disease were found to have a higher probability of upstaging at RC specimen and to have a decreased disease-specific and progression-free survival probabilities compared to low-risk patients. These results have been recently validated in an external cohort [36]. Indeed, this clinical preoperative risk grouping might help the decision-making process regarding the administration of NAC [35, 36]. The most important studies of this paragraph are summarized in Table 2.

**Table 2:** Selected studies analyzing the clinical characteristics predicting recurrence in patients treated with open radical cystectomy for bladder cancer

First author and year	Number of patients	Median age (years)	NAC (%)	Clinical tumor stage	Pathologic tumor stage	Pathologic node stage	Adjuvant therapies (%)	Median follow-up (months)	Survival	Predictive factors associated with recurrence and other findings
D’Andrea et al. [19], 2017	4335	67	0	≤ cT1: 36% ≥ cT2: 64%	pT0: 5% pTa: 3% pTis: 10% pT1: 14% pT2: 24% pT3: 31% pT4: 13%	pN+: 26%	23	42.4	5-year RFS 77.7% 5-year RFS in: NLR ≥ 2.7: 54% NLR < 2.7: 67% 5-year RFS in: LMR < 3.5: 52% LMR ≥ 3.5: 60%	At multivariable analysis controlling for the effects of standard clinicopathologic variables, **LMR (HR 1.4;** ***p*** **<** **0.001**) and **NLR (HR 1.2;** ***p*** **<** **0.001)** were independently associated with RFS
Viers et al. [20], 2014	899	69	0	≤ cT1: 41% cT2: 53% cT3/4: 6%	≤ pT1: 44% pT2: 17.5% pT3: 32% pT4: 6%	pN0: 73.1% pN1: 6.6% pN2: 8.1% pN3: 2% pNx: 10.1%	13	130	10-year RFS 65.5%	Increased preoperative **NLR analyzed as continuous variable (HR 1.04;** ***p*** **=** **0.02)** was independently associated with greater risks of RFS
Djaladat et al. [22], 2014	1471	< 70: 57.9% ≥ 70: 42.1%	7.3	NR	≤ pT2 N0: 56% > pT2 N0: 20% pT any N+: 24%	pN+: 24%	22.4	148.8	5-year RFS in: Serum albumin < 3.5 g/dL: 53.1% Serum albumin ≥ 3.5 g/dL: 68.6%	Adjusting for multiple potential confounding factors, **low serum albumin level** (< 3.5 g/dL) was independently associated with both decreased OS (HR 1.93, *p* < 0.001) and RFS (**HR 1.68,** ***p*** **=** **0.006**)
Chromecki et al. [2] 2014	4118	67	0	NR	pT0: 5% pTa: 3% pTis: 10% pT1: 14% pT2: 24% pT3: 31% pT4: 13%	pN+: 25%	22.1	134.4	3-year RFS: 63% 7-year RFS: 60% 10-year RFS: 57%	**BMI** **>** **30** coded as a categorical variable was an independent predictor of disease recurrence **(HR 1.67,** ***p*** **<** **0.0001)** cancer-specific mortality (HR 1.43, *p* < 0.001), and overall mortality (HR 1.81, *p* < 0.001). The same was true for BMI when analyzed as a continuous variable (all *p* values < 0.001)
Rieken et al. [32] 2014	1504	66	0	NR	pT0: 5% pTa: 4% pTis: 11% pT1: 11% pT2: 27% pT3: 31% pT4: 11%	pN+: 22%	21.4	34	3-year RFS: 66% 3-year RFS in: Nondiabetics 65% DM without metformin use: 53% DM with metformin use: 62%	Metformin use (HR: 0.61, *p* = 0.04) was associated with decreased risk of disease recurrence, while DM without metformin use (HR 1.40, *p* = 0.02) was associated with increased risk of disease recurrence at univariable analysis In multivariable Cox regression analyses **DM with (HR: 0.96,** ***p*** **=** **0.96) or without metformin use (HR: 1.29,** ***p*** **=** **0.08)** was not associated with disease recurrence
Culp et al. [35] 2014	297	High-risk clinical disease: 72.6 years Low-risk clinical disease: 69.6 years	0	cT1: 9.8% cT2: 83.8% cT3: 4.7% cT4: 1.7%	pT0: 8.8% pTis: 11.5% pTa: 3.7% pT1: 5.4% pT2: 23.2% pT3: 41% pT4: 6.4%	pN+: 25.3%	17.1	40.6	NR	At competing risk analysis, after adjusting for age at surgery, gender, race, preoperative anemia, year of surgery and smoking history, high-risk disease was significantly associated with worse 5-year DSS (68.2% vs 82.7%, *p* < 0.001) and PFS (63.9% vs 83.6%, *p* < 0.001) compared to low-risk disease
Moschini et al. [36] 2017	343	High-risk clinical disease: 65 years Low-risk clinical disease: 66 years	0	≤ cT2: 86.9% cT3: 7.9% cT4: 5.2%	pTis: 2.3% pTa: 1.7% pT1: 3% pT2: 20.2% pT3: 50.4% pT4: 22.4%	pN+: 43%		160	Recurrence rate 46%	After adjusting for age at surgery, gender and year of surgery, patients with high-risk clinical disease had a lower 5-year DFS (64.4% vs 77.4%) and 5-year PFS (67.1% vs 45.8%) compared to patients with low-risk clinical disease

Bold highlights the risk related to recurrence for each of the factors included

*CIS* carcinoma in situ, *DM* diabetes mellitus, *DFS* disease-free survival, *DSS* disease-specific survival, *LMR* lymphocyte-to-monocyte ratio, *NAC* neoadjuvant chemotherapy, *NLR* neutrophil-to-lymphocyte ratio, *NR* not reported, *PFS* progression-free survival, *RFS* recurrence-free survival

#### Histologic patterns

The prognostic value of pathologic features on recurrence is of great importance in patients treated with open RC. Lotan et al. [37] reported that LVI was an independent predictor of local, distant and overall recurrence in pN0 patients treated with open RC. Several studies unanimously confirmed that LVI at RC specimen has an independent association with RFS after surgery. In a report of 2000 patients, variant histology (VH) of urothelial BC at bladder specimen accounts for approximately 25% of cases of patients undergoing RC [38]. Patients with VH had a significantly higher disease recurrence rate compared to those with pure urothelial BC patients or with squamous-cell differentiated BC. Nonetheless, this effect did not remain significant on the multivariable model [38]. Soave et al. [39] demonstrated that the presence of VH and non-squamous cell differentiation had not a significant impact on oncological outcomes. Similarly, Fairey et al. demonstrated that micropapillary variant of urothelial BC had similar oncological outcomes after open RC to those with pure urothelial BC after controlling for relevant clinical and pathologic factors [40]. Recently, Moschini et al. reported that pure VH, mostly represented by small-cell and micropapillary VH, compared to the pure urothelial BC at RC specimen was an independent predictor of CSM and RFS [41]. In a cohort of patients with cT1-4N0-2 treated with cisplatin-based NAC and RC, the squamous or glandular VH was an independent predictor of pathologic downstaging [42]. Furthermore, these patients presenting squamous or glandular VH had comparable overall survival compared to those with pure urothelial BC.

Contrasting results were reported regarding the impact of carcinoma in situ (CIS) at RC specimen on recurrence-free survival. In a retrospective evaluation, the clinical data of 812 patients treated with RC were analyzed [43, 44]. The presence of concomitant CIS (46% of cases) was significantly associated with urethra involvement at the pathologic specimen and, in patients with organ-confined disease, was independently associated with disease recurrence, but not bladder CSM [44]. Patients with CIS only at RC (12%) had no LN metastasis with durable control even in the case of failure after intravesical therapy [43]. The concomitant CIS was found to be an independent predictor of upper urinary tract carcinoma [45] Conversely in two other studies, concomitant CIS was not independently associated with survival outcomes [46, 47]. A recent analysis of 1128 patients undergoing RC without NAC evaluated the impact of CIS on survival outcomes [29]. The presence of CIS was independently associated with CSM only in the subcohort of patients harboring an organ-confined disease. Patients with concomitant CIS had a significantly increased risk of developing urothelial recurrences regardless of the tumor stage [29]. The most important studies of this paragraph are summarized in Supplementary Table 2.

#### Pathologic patterns, tumor and node stage

Positive STSMs seem to be significantly higher in patients with a non-organ-confined disease compared to ≤ pT2 cases despite the surgical volume of the center [48, 49]. In a retrospective single-center study evaluating oncologic outcomes of 1589 patients treated with open RC, Dotan et al. showed that the most common locations were the posterior and lateral walls of the bladder and the periprostatic soft tissue [49]. RFS was significantly higher in patients with positive compared to those with negative STSMs. The positive STSMs were independently associated with disease-specific death, but not with metastatic progression after adjusting for clinicopathological factors [49]. Patients with urethral positive surgical margin had an 18-fold higher risk of urethral recurrence. In a multicentre overview of 154 patients with positive STSMs after RC, the local RFS and CSM was significantly lower in patients with positive urethral surgical margin [50]. However, at multivariable analysis the urethral margin was not an independent predictor of CSM.

Tumor and node stage at pathologic evaluation represent two powerful predictors of recurrence and CSM [1]. The International Bladder Cancer Nomogram Consortium analyzed the oncologic outcomes of 9064 patients treated with RC and lymphadenectomy, including 1550 patients with LN involvement [51]. The Authors developed a postoperative nomogram to predict the risk of recurrence. The pathologic tumor stage and grade and node status were found to have a direct correlation with the risk of recurrence. This association was confirmed in other nomograms developed in wide international series [52, 53].

In a single-center retrospective study enrolling 1388 patients, Umbreit et al. evaluated a multi-factorial, site-specific recurrence model in patients treated with open RC [54]. The abdominal/pelvic, upper urinary tract (UUT), thoracic and bone recurrence rate was 4.8, 28, 10.3 and 11%, respectively. After adjusting for clinicopathological features, pT4 stage, positive ureteral margins and multifocality were found to be independent predictors of upper tract recurrence. pT3 and pT4 stage, lymph node invasion and multifocality were found to be independent predictors of abdominal/pelvic, thoracic and bone recurrence.

In a retrospective study involving 1600 patients treated with open RC at a tertiary referral center, extravesical tumor extension and LN density (ratio positive/total node at pathology) greater than 4% were significantly associated with a higher recurrence rate [55]. Conversely, Tarin et al. analyzed the oncologic outcomes of 591 patients treated with open RC and PLND [56]. After adjusting for perioperative features, the number of positive LN (none, 1, or 2 or more) was significantly associated with CSM. LN density was not a significant predictor of recurrence or CSS. RFS or CSS did not differ significantly between pN1, pN2, and pN3. Simone et al. [57] retrospectively identified the LN density cut-off values to predict CSS in 156 pN+ patients treated with RC and PLND. The thresholds were 11 and 30% for LN density, 9 and 30 for LN count. In the present study, PLND was the greatest predictor of CSM.

Contrasting studies were reported regarding the influence of PLND extension on LN stage [57, 58]. However, the standard field of PLND should include the lymphatic tissue around the common iliac, external iliac, internal iliac artery, and obturator region. As crossover lymphatic drainage is very common, PLND should always be performed bilaterally [1].

Several studies hypothesized that an extended PLND (ePLND) up to the aortic bifurcation or even super-extended PLND (sePLND) up to the inferior mesenteric artery could be associated with better survival. May et al. [59] showed that the tumor-specific survival and the disease-free interval were improved when an ePLND with equal to or more than 16 removed lymph nodes was performed. Zehnder et al. [60] conducted a retrospective bi-center cohort study on patients who underwent open RC and PLND with curative intent for pT2-3cN0M0 BC. In one institution, all patients were treated with an ePLND up to the mid-upper third of the common iliac vessels. In the other institution, all patients were treated with a sePLND. At multivariable analysis, after adjusting for Institution, pathological subgroup and AC, LN status and number of positive LNs were independent risk factors for RFS. The authors concluded that meticulous ePLND appears to provide survival and recurrence outcomes similar to those of sePLND [60]. However, no evidence is reported regarding the possible oncologic benefits of sePLND in particular classes of patients such as those with a clinically suspicious LN involvement. The most important studies of this paragraph are summarized in Supplementary Table 3.

## Conclusions

A significant proportion of patients relapse after RC for MIBC. Local recurrence rate ranges between 30 and 54%. Distant recurrence is not often standardized and is reported in up to 50% of cases.

Higher age, female gender, and smoking exposure at the time of RC are the most relevant epidemiological predictors of recurrence after RC. Preoperative higher NLR and lower hemoglobin are significantly associated with a worse RFS. Obesity is an independent predictor of RFS. The presence of micropapillary, neuroendocrine features and LVI at TUR specimen, hydroureteronephrosis and cT3b-T4a disease are clinical factors defining a high-risk disease, and are independent predictors of RFS after RC. Among the histologic patterns, LVI, pure VH and concomitant CIS have an independent correlation with RFS after open RC. Advanced tumor stage, multifocality, positive STSMs and LN involvement are the most relevant pathologic predictors of RFS after RC. A limited PLND resulted to be significant intraoperative predictors of recurrence. Better oncologic outcomes were reported in patients submitted to ePLND, while a sePLND seemed to not provide a significantly lower recurrence rate.

## Electronic supplementary material

Below is the link to the electronic supplementary material.

Supplementary material 1 (DOCX 17 kb)

Supplementary material 2 (DOCX 30 kb)

Supplementary material 3 (DOCX 28 kb)
