# Supplementary material for: Patterns and predictors of recurrence after open radical cystectomy for bladder cancer: a comprehensive review of the literature
Source: World J Urol. 2017 Nov 16;36(2):157–70. doi: 10.1007/s00345-017-2115-4 (PMC5799348; doi:10.1007/s00345-017-2115-4)
Supplement: Supplementary file 1 — Supplementary material 1 (DOCX 17 kb) [file 345_2017_2115_MOESM1_ESM.docx]

| **First author** | **Journal (year)** | **Random sequence generation (selection bias)** | **Allocation concealment (selection bias)** | **Blinding of participants and personnel (performance bias)** | **Blinding of outcome assessment (detection bias)** | **Incomplete outcome data (attrition bias)** | **Selective reporting (reporting bias)** | **Other biases** |
| --- | --- | --- | --- | --- | --- | --- | --- | --- |
| Bellmunt J | Am J Clin Oncol (1997) |  |  |  |  |  |  |  |
| Hellsten S | Eur Urol (1998) |  |  |  |  |  |  |  |
| International collaboration of trialists | Lancet (1999) |  |  |  |  |  |  |  |
| Kaufman DS | Oncologist (2000) |  |  |  |  |  |  |  |
| Millikan R | J Clin Oncol (2001) |  |  |  |  |  |  |  |
| Sherif A | Scand J Urol Nephrol (2002) |  |  |  |  |  |  |  |
| Sengeløv L | Acta Oncol (2002) |  |  |  |  |  |  |  |
| Grossman HB | N Engl J Med (2003) |  |  |  |  |  |  |  |
| Lotan Y | J Clin Oncol (2005) |  |  |  |  |  |  |  |
| Bochner BH | J Clin Oncol (2006) |  |  |  |  |  |  |  |
| Karakiewicz PI | J Urol (2006) |  |  |  |  |  |  |  |
| Koppie TM | Cancer (2008) |  |  |  |  |  |  |  |
| Ghoneim MA | J Urol (2008) |  |  |  |  |  |  |  |
| Bruins HM | J Urol (2009) |  |  |  |  |  |  |  |
| deVere White RW | J Urol (2009) |  |  |  |  |  |  |  |
| Isbarn H | J Urol (2009) |  |  |  |  |  |  |  |
| Shariat SF | BJU Int (2010) |  |  |  |  |  |  |  |
| Griffiths G | J Clin Oncol. (2011) |  |  |  |  |  |  |  |
| Cognetti F | Ann Oncol (2012) |  |  |  |  |  |  |  |
| Hautmann RE | Eur Urol (2012) |  |  |  |  |  |  |  |
| Abdollah F | Ann Surg Oncol (2012) |  |  |  |  |  |  |  |
| Chromecki TF | BJU Int (2013) |  |  |  |  |  |  |  |
| Horovitz D | BJU Int (2013) |  |  |  |  |  |  |  |
| Rink M | Eur Urol (2013) |  |  |  |  |  |  |  |
| Christodouleas JP | Cancer (2014) |  |  |  |  |  |  |  |
| Khaled HM | Clin Genitourin Cancer (2014) |  |  |  |  |  |  |  |
| Choueiri TK | J Clin Oncol (2014) |  |  |  |  |  |  |  |
| Plimack ER | J Clin Oncol (2014) |  |  |  |  |  |  |  |
| Kitamura H | Ann Oncol (2014) |  |  |  |  |  |  |  |
| Ohyama C | Int J Urol (2014) |  |  |  |  |  |  |  |
| Messer JC | Urology (2014) |  |  |  |  |  |  | * |
| MItra AP | Urol Oncol (2014) |  |  |  |  |  |  |  |
| Viers BR | Eur Urol (2014) |  |  |  |  |  |  |  |
| Hermanns T | Br J Cancer (2014) |  |  |  |  |  |  |  |
| Djaladat H | BJU Int (2014) |  |  |  |  |  |  |  |
| Moschini M | Anticancer Res (2014) |  |  |  |  |  |  |  |
| May M | Clin Genitourin Cancer (2014) |  |  |  |  |  |  |  |
| Culp SH | J Urol (2014) |  |  |  |  |  |  |  |
| Linder BJ | J Urol (2014) |  |  |  |  |  |  |  |
| Koie T | Med Oncol (2015) |  |  |  |  |  |  |  |
| Sternberg CN | Lancet Oncol (2015) |  |  |  |  |  |  |  |
| Engel O | World J Urol (2015) |  |  |  |  |  |  |  |
| Salama A | Clin Genitourin Cancer (2016) |  |  |  |  |  |  |  |
| Hahn NM | Urol Oncol (2016) |  |  |  |  |  |  |  |
| Morizawa Y | Urol Oncol (2016) |  |  |  |  |  |  | ** |
| Dabi Y | World J Urol (2017) |  |  |  |  |  |  |  |
| D’Andrea D | J Surg Oncol (2017) |  |  |  |  |  |  | *** |
| Soria F | Urology (2017) |  |  |  |  |  |  | **** |

Supplementary Table 1: Risk of bias assessment tool of the 48 studies included to investigate recurrence patterns. Green indicates low risk of bias, yellow unclear, and red high risk of bias.

*Other biases: Insufficient numbers of patients within substages. **Small sample size (110 pts). *** Patients’ accrual over a period of almost 15 years. **** No standardization of follow-up.

**World Journal of Urology®**

**Patterns and predictors of recurrence after open radical cystectomy for bladder cancer: a Comprehensive Review of the literature.**

Andrea Mari^1,2^, Riccardo Campi^1^, Riccardo Tellini^1^, Giorgio Gandaglia^3^, Simone Albisinni^4^, Mohammad Abufaraj^2, 5^, Georgios Hatzichristodoulou^6^, Francesco Montorsi^3^, Roland van Velthoven^4^, Marco Carini^1^, Andrea Minervini^1^, Shahrokh F. Shariat^2,7,8,9^.

**Affiliations:**

^1^ Department of Urology, University of Florence, Careggi Hospital, Florence, Italy.

^2^ Department of Urology, Medical University of Vienna, Vienna, Austria.

^3^ Division of Oncology/Unit of Urology, IRCCS San Raffaele Hospital, URI, Milan, Italy.

^4^ Department of Urology, Institut Jules Bordet, Université Libre de Bruxelles, Belgium

^5^ Division of Urology, Department of Special Surgery, Jordan University Hospital, The University of Jordan, Amman, Jordan.

^6^ Department of Urology and Pediatric Urology, Julius-Maximilians-University of Würzburg, Würzburg, Germany.

^7^ Karl Landsteiner Institute of Urology and Andrology, Vienna, Austria.

8 Department of Urology, University of Texas Southwestern Medical Center, Dallas, TX, USA.

9 Department of Urology, Weill Cornell Medical College, New York, NY, USA.

**Corresponding author:**

Shahrokh F. Shariat. Department of Urology and Comprehensive Cancer Center, Vienna General Hospital, Medical University of Vienna. Email: shahrokh.shariat@meduniwien.ac.at.
