# Supplementary material for: Patterns and predictors of recurrence after open radical cystectomy for bladder cancer: a comprehensive review of the literature
Source: World J Urol. 2017 Nov 16;36(2):157–70. doi: 10.1007/s00345-017-2115-4 (PMC5799348; doi:10.1007/s00345-017-2115-4)
Supplement: Supplementary file 2 — Supplementary material 2 (DOCX 30 kb) [file 345_2017_2115_MOESM2_ESM.docx]

| **First author and year** | **Number of patients** | **Median age (years)** | **NAC** | **Pathologic Tumor stage** | **Pathologic Node stage** | **LVI (%)** | **VH (%)** | **Concomitant CIS (%)** | **Median follow-up (months)** | **Survival** | **Predictive factors associated with recurrence and other findings** |
| --- | --- | --- | --- | --- | --- | --- | --- | --- | --- | --- | --- |
| Lotan et al. [37] 2005 | 750 | 65.9 | 4.9% | pTis-1: 24%  pT2: 34.8%  pT3: 29.3%  pT4: 11.9% | pN0: 77.5%  pN1: 8.8%  pN2: 12%  pN3: 1.7% | 36.4% | NR | 7.5% | 37 | NR | **LVI** was an independent predictor of **local (HR: 2.03; p=0.049), distant (HR: 2.60; p=0.0011) and overall recurrence (HR: 2.02; p=0.003)** in node-negative patients. |
| Xylinas et al. [38] 2013 | 488 | 67 | 0% | pTa: 3%  pTis: 11%  pT1: 17%  pT2: 26%  pT3: 31%  pT4: 12% | pN+: 21% | 36.3% | PU 75.4%  VH 24.6% | 59% | 55 | 3-yr RFS: 65%  5-yr RFS: 59%  10-yr RFS: 56% | After adjusting for age, gender, pT3-4 and grade, pN+, concomitant CIS, LVI and STSM, none of the VH was independently associated with disease recurrence and CSS. In patients treated with AC (n = 492) there was no difference in CSS between pure UCB, squamous cell differentiated UC and other histological UC variants. |
| Soave et al. [39] 2015 | 485 | 66 | 0% | pTa: 5%  pTis: 12%  pT1: 15%  pT2: 23%  pT3: 27%  pT4: 18% | 28.5% | 31.5% | 20% | 40.6% | 45 | 2-yr RFS: 68%  5-yr RFS: 58% | At univariable analysis VH vs PU BCa (HR: 1.44; p=0.05), squamous cell differentiation vs. PU BCa (HR: 1.12; p=0.66), non-squamous cell differentiation vs. PU BCa (HR: 2.14; p=0.003) were differently associated with disease recurrence. At multivariable analysis predicting **neither** the presence of **VH** **nor non-squamous cell differentiation nor the extent of VH were associated with RFS.** |
| Fairey et al. [40] 2014 | 1380 | 68 (TCC)  67 (MP) | 7% [TCC];  6% [MP] | ≤pT2N0M0: 91%  ≥pT3N0M0: 9% | Tany N1–3 M0: 2.4% | 29.3% | MP 2.4% | 62.2% | 120 | 5-yr RFS: 69% [TCC];  5-yr RFS: 58% [MP] | **Pathologic TNM stage (p<0.01), LVI (HR: 1.6; p<0.01), histologic grade (HR: 1.65; p<0.01) and adjuvant chemotherapy (HR: 0.56; p<0.01)** were all **significantly associated with RFS**, while histologic type was not independently associated with RFS (p=0.92). |
| Moschini et al. [41] 2016 | 1067 | 68 | 2.7% | pT0-T2: 38%;  pT3: 41%;  pT4: 21% | pN+: 36% | 26.1% | PU 68%  MV 19%  PV 13% | 25% | 74 | NR | Pure versus urothelial variants were found to be related to worse survival considering recurrence (HR: 2.04; p<0.001), CSM and OM. Conversely, the presence of mixed variant was not associated with survival outcomes (all p>0.2). |
| Shariat et al. [44] 2007 | 330 | 66.4 | 6% | pTa-1: 24.5%  pT2: 31.5%  pT3: 30.3%  pT4: 13.7% | pN+: 27.6% | 41.2% | TCC | 100% | 36.4 | 3-yr RFS: 59%  5-yr RFS: 55.1%  7-yr RFS: 50.3% | In patients with organ-confined disease, concomitant CIS was an independent predictor of disease recurrence (HR: 1.92; p=0.012). |
| Shariat et al. [43] 2006 | 99 | 66.1 | 2% | CIS (100%) | pN+: 3% | 2% | TCC | 0% (CIS only 100%) | 39.2 | 3-yr RFS: 89.8%  5-yr RFS: 83%  7-yr RFS: 83% | At univariate Cox regression  analyses, only metastasis to regional lymph nodes was associated with disease recurrence (HR: 17.8; p<0.001). |
| Yafi et al. [47] 2014 | 1968 | 68 |  | ≤pT2 48%  pT3-4 52% | pN+ 26% |  | Non-UC 6.2% | 19.8% | 29 |  | In patients with cTa-1 BCa disease, the pT>2 (HR 2.77; p<0.001) and pN+ (HR: 2.25; p<0.001), but not concomitant CIS (HR: 2.25; p<0.001) and non-UC (HR: 1.44 p=0,18), were independent predictors of RFS. In patients with cT2 BCa disease, non-UC (HR: 2.92 p=0,01), the pT>2 (HR 2.29; p=0.001) and pN+ (HR: 2.01; p=0.003), but not concomitant CIS (HR: 1.23; p=0.31), were independent predictors of RFS |
| Moschini et al. [29] 2015 | 1128 | 68 | 0% | pT0-2: 40.5%  pT3: 38.8%  pT4: 20.7% | pN0: 63.5%  pN1: 10.7%  pN2: 22%  pN3: 3.8% | 8.4% | TCC | 24.6% | 72 | 5-yr RFS in:  pT0N0: 79%  pT0-2 without CIS: 73%  pT0-2 with CIS: 74%  5-year pT3-4 without CIS RFS: 41.3%  5-year pT3-4 with CIS RFS: 40.1% | At multivariable analysis **CCI** (**HR: 1.41; p=0.01), positive margins (HR: 1.74; p=0.02), pN+ (HR: 1.53; p=0.01), number of positive LN (HR: 1.03; p=0.003) and AC (HR: 1.37; p=0.04) were independent predictors of RFS.** Concomitant CIS was not associated with any survival effect when the overall population was considered. the presence of CIS was associated with worse CSM in pT0–pT2 patients only (HR: 1.82; p=0.04). |
| AC: Adjuvant chemotherapy; BCa: bladder cancer; CSS: cancer specific survival; LVI: lymphovascular invasion; MP: micropapillary; NAC neoadjuvant chemotherapy; NR: not reported; OM: overall mortality; TCC: transitional cell carcinoma; UC: urothelial carcinoma; VH: Variant histology | | | | | | | | | | | |

Supplementary table 2: Selected studies analyzing the histologic patterns predicting recurrence in patients treated with open radical cystectomy for bladder cancer.

**World Journal of Urology®**

**Patterns and predictors of recurrence after open radical cystectomy for bladder cancer: a Comprehensive Review of the literature.**

Andrea Mari^1,2^, Riccardo Campi^1^, Riccardo Tellini^1^, Giorgio Gandaglia^3^, Simone Albisinni^4^, Mohammad Abufaraj^2, 5^, Georgios Hatzichristodoulou^6^, Francesco Montorsi^3^, Roland van Velthoven^4^, Marco Carini^1^, Andrea Minervini^1^, Shahrokh F. Shariat^2,7,8,9^.

**Affiliations:**

^1^ Department of Urology, University of Florence, Careggi Hospital, Florence, Italy.

^2^ Department of Urology, Medical University of Vienna, Vienna, Austria.

^3^ Division of Oncology/Unit of Urology, IRCCS San Raffaele Hospital, URI, Milan, Italy.

^4^ Department of Urology, Institut Jules Bordet, Université Libre de Bruxelles, Belgium

^5^ Division of Urology, Department of Special Surgery, Jordan University Hospital, The

University of Jordan, Amman, Jordan.

^6^ Department of Urology and Pediatric Urology, Julius-Maximilians-University of Würzburg, Würzburg, Germany.

^7^ Karl Landsteiner Institute of Urology and Andrology, Vienna, Austria.

8 Department of Urology, University of Texas Southwestern Medical Center, Dallas, TX, USA.

9 Department of Urology, Weill Cornell Medical College, New York, NY, USA.

**Corresponding author:**

Shahrokh F. Shariat. Department of Urology and Comprehensive Cancer Center, Vienna General Hospital, Medical University of Vienna. Email: shahrokh.shariat@meduniwien.ac.at.
