# Supplementary material for: Patterns and predictors of recurrence after open radical cystectomy for bladder cancer: a comprehensive review of the literature
Source: World J Urol. 2017 Nov 16;36(2):157–70. doi: 10.1007/s00345-017-2115-4 (PMC5799348; doi:10.1007/s00345-017-2115-4)
Supplement: Supplementary file 3 — Supplementary material 3 (DOCX 28 kb) [file 345_2017_2115_MOESM3_ESM.docx]

| **First author and year** | **Number of patients** | **Median age (years)** | **NAC** | **Pathologic tumor stage** | **Pathologic node stage** | **Surgical margins** | **Adjuvant therapies** | **Median follow-up (months)** | **Survival** | **Predictive factors associated with recurrence and other findings** |
| --- | --- | --- | --- | --- | --- | --- | --- | --- | --- | --- |
| Dotan et al. [49] 2007 | 1589 | ≥65 yrs 61% | 11% | ≤pT2: 54%  pT3-4: 46% | pN+: 24% | 4.2% | 17% | NR | 5-yr DSS 71%  10-yr DSS 66% | **pT3-4 (HR: 1.96; p<0.001), LVI (HR 1.73), positive STSM (HR: 1.98, p=0.01), pN+ (HR: 1.88; p<0.001), number of LN removed (HR: 0.97; p=0.001) and number of LN removed (HR 1.06, p=0.03)** were independent predictors of **recurrence.** |
| Bochner et al. [51] | 9064 | NR | 0% | NR | NR | NR | 0% | 30 | NR | The nomogram predicting risk of recurrence after RC included information on patient age, sex, time from diagnosis to surgery, pathologic tumor stage and grade, tumor histologic subtype, and regional lymph node status (concordance index, 0.75) |
| Umbreit et al. [54] 2010 | 1388 | 68 | 2% | pT0-1: 32%  pT2: 37%  pT3: 22%  pT4: 9% | pNX :12%  pN0, 1-10 LN: 31%  pN0, >11 LN: 45%  pN1/pN2: 12% | STSM 1%  Positive urethral margin 3%  Positive ureteral margin 2% | 5% | 182 | Overall RFS was NR | Predictors of **Upper Urinary Tract recurrenc**e: pT4 stage (HR: 2.84), positive ureteral margins (HR: 5.71), multifocality (HR: 2.07) (all p<0,01). Predictors of **Abdominal/Pelvic recurrence**: pT3 (HR: 2.30) and pT4 (HR: 3.55), pNx (HR: 1.66) pN+ (HR: 1.97), 1-10 LN removed (HR 1.52), multifocality (HR: 1.80) (all p<0.01), prostatic involvement (HR: 1.45; p=0.019). Predictors of **Thoracic recurrence**: pT3 (HR: 2.61) and pT4 (HR: 3.39), pN+ (HR: 2.64), multifocality (HR: 1.79) (all p<0,01), (HR: 1.89; p=0.02), 1-10 LN removed (HR: 1.58; p<0.03),. Predictors of **Bone recurrence**: pT3 (HR: 3.45) and pT4 (HR: 3.87), pN+ (HR: 1.79), radiation exposure (HR: 2.97) (all p <0.01), positive urethral margin (HR: 2.28; p=0.039). |
| Bruins et al. [55] 2009 | 181 pN+ | 66 | 5% | ≤pT1: 11.7%  pT2a: 9.9%  pT2b: 21%  pT3a: 15.5%  pT3b: 29.8%  pT4: 12.2% | -pN0-x: 0%  -1 positive lymph node: 65%;  -2 positive lymph nodes: 35%. | 2% | 97%  (3% Adj RT) | 153 | 5-year RFS: 43.8%  10-year RFS: 40.9% | **Pathological stage/subgroup (HR: 1.733; p=0.015), LN density >4% (HR: 1.935; p=0.014)** **and adjuvant chemotherapy (HR: 0.538; p=0.004)** were significant independent predictors of **RFS**. |
| Tarin et al. [56] 2012 | 591 | 66 | 23% | pT0-1: 53%  pT2: 15%  pT3: 29%  pT4: 3% | pN0: 80%  pN1: 9%  pN2: 3%  pN3: 7% | 1.5% | 15% | 30 | 5-year pN0 RFS: 69%  5-year pN+ RFS: 33% | **>2 positive nodes (HR: 3.62; p<0.0005) and >pT3 stage (HR: 2.94; p<0.0005)** were significantly associated with disease recurrence, while positive lymph node location was not an independent predictor of RFS (p=0.5). |
| Simone et al [57] 2012 | 156 (pN+ pts after RC + LND) | 67 | 0% | pT0-a-is-1: 7%  pT2: 11%  pT3: 53%  pT4: 29% | pN1: 22%  pN2: 55%  pN3: 23% | NR | 28%  (2% Adj RT) | 24 | NR | NR |
| Zehnder et al. [60] 2011 | 959 | 67 | 0% | pT2: 44%  pT3: 52.6% | pN+: 32% | 0% | 25% | 120 | 5-yr RFS in pN0:  -ePLND 59%  -sePLND 71%  5-yr RFS in pN0:  -ePLND 40%  -sePLND 42% | Pathological subgroup (pT3 vs pT2), LN status and number of positive LNs were independent risk factors for RFS and OS while institution, age and adjuvant chemotherapy predicted only OS (HR were not reported).  All cases where performed in two different institutions performing systematically sePLND and ePLND, respectively. The institution was not an independent predictor of RFS or OS. |
| LVI lymphovascular invasion; LND lymph node dissection (e: extended; se: superextended); LVI: lymphovascular invasion; MP: micropapillary; NAC neoadjuvant chemotherapy; NR: not reported; OM: overall mortality; RC: radical cystectomy; RT radiotherapy; STSM: soft-tissue surgical margins; TCC: transitional cell carcinoma; VH: Variant histology | | | | | | | | | | |

Supplementary table 3: Selected studies analyzing the role of pathologic patterns associated with recurrence in patients treated with open radical cystectomy for bladder cancer.

**World Journal of Urology®**

**Patterns and predictors of recurrence after open radical cystectomy for bladder cancer: a Comprehensive Review of the literature.**

Andrea Mari^1,2^, Riccardo Campi^1^, Riccardo Tellini^1^, Giorgio Gandaglia^3^, Simone Albisinni^4^, Mohammad Abufaraj^2, 5^, Georgios Hatzichristodoulou^6^, Francesco Montorsi^3^, Roland van Velthoven^4^, Marco Carini^1^, Andrea Minervini^1^, Shahrokh F. Shariat^2,7,8,9^.

**Affiliations:**

^1^ Department of Urology, University of Florence, Careggi Hospital, Florence, Italy.

^2^ Department of Urology, Medical University of Vienna, Vienna, Austria.

^3^ Division of Oncology/Unit of Urology, IRCCS San Raffaele Hospital, URI, Milan, Italy.

^4^ Department of Urology, Institut Jules Bordet, Université Libre de Bruxelles, Belgium

^5^ Division of Urology, Department of Special Surgery, Jordan University Hospital, The University of Jordan, Amman, Jordan.

^6^ Department of Urology and Pediatric Urology, Julius-Maximilians-University of Würzburg, Würzburg, Germany.

^7^ Karl Landsteiner Institute of Urology and Andrology, Vienna, Austria.

8 Department of Urology, University of Texas Southwestern Medical Center, Dallas, TX, USA.

9 Department of Urology, Weill Cornell Medical College, New York, NY, USA.

**Corresponding author:**

Shahrokh F. Shariat. Department of Urology and Comprehensive Cancer Center, Vienna General Hospital, Medical University of Vienna. Email: shahrokh.shariat@meduniwien.ac.at.
